# Supplementary material for: Micromachined structures decoupling Joule heating and electron wind force
Source: Nat Commun. 2024 Jul 18;15:6044. doi: 10.1038/s41467-024-50351-8 (PMC11258259; doi:10.1038/s41467-024-50351-8)
Supplement: Supplementary file 1 — Supplementary Information [file 41467_2024_50351_MOESM1_ESM.pdf]

Supplementary Information for

Micromachined Structures Decoupling Joule Heating and Electron Wind Force

Shaojie Gu[a,*], Yasuhiro Kimura[a], Xinming Yan[a], Chang Liu[a], Yi Cui[b], Yang Ju[c,*], Yuhki Toku[a,*]

[a] Department of Micro-Nano Mechanical Science and Engineering, Graduate School of Engineering, Nagoya University, Nagoya, 464-8601, Japan

[b] Department of Mechanical Systems Engineering, Graduate School of Engineering, Nagoya University, Nagoya, 464-8601, Japan

[c] School of Mechanical Engineering, Zhejiang University, Hangzhou, 310027, China

* Corresponding authors' emails: gu.shaojie.e7@f.mail.nagoya-u.ac.jp (Shaojie Gu), yang.ju@zju.edu.cn (Yang Ju), toku@nagoya-u.jp (Yuhki Toku)

The PDF file includes:

- Supplementary Figures 1–10
- Supplementary Tables 1–2
- Supplementary Notes 1–2
- Supplementary References

Supplementary Figures

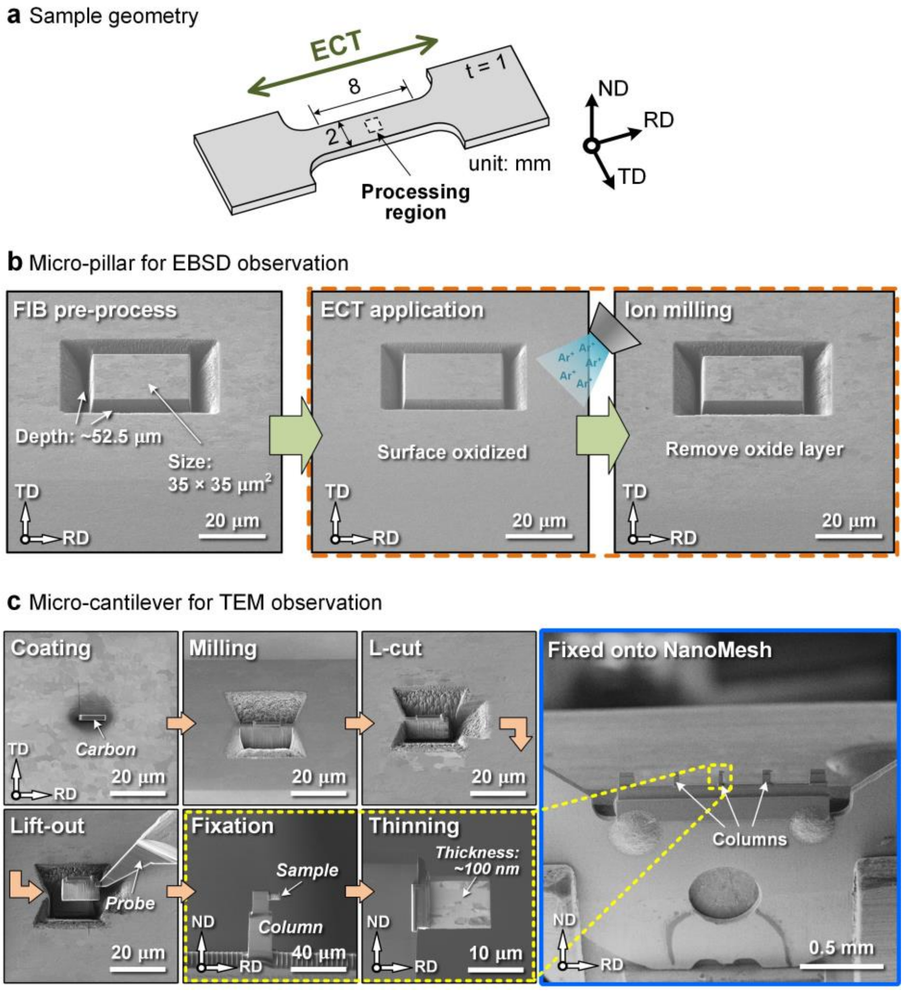

Supplementary Figure 1 | Sample geometry and micromachining. (a) Geometry of the sample (unit: mm), where the tensile and ECT apply along the rolling direction (RD). **(b)** Micromachined pillar for EBSD observation before and after ECT and the ion milling method used to remove the oxide layer caused by ECT. **(c)** Micromachined cantilever for TEM observation and the FIB processing including coating, milling, L-cut, lift-out, fixation, and thinning. The extracted thin sample is fixed onto a column of a NanoMesh for TEM observation.

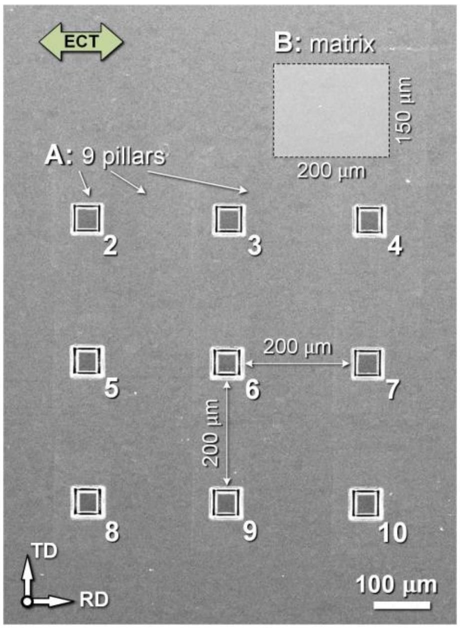

Supplementary Figure 2 | Additionally processed nine micro-pillars (No. 2–No. 10) and a larger matrix observation area (200 µm × 150 µm). The spacing between the pillars was set to 200 µm to prevent the influence of electric current bypassing between them.

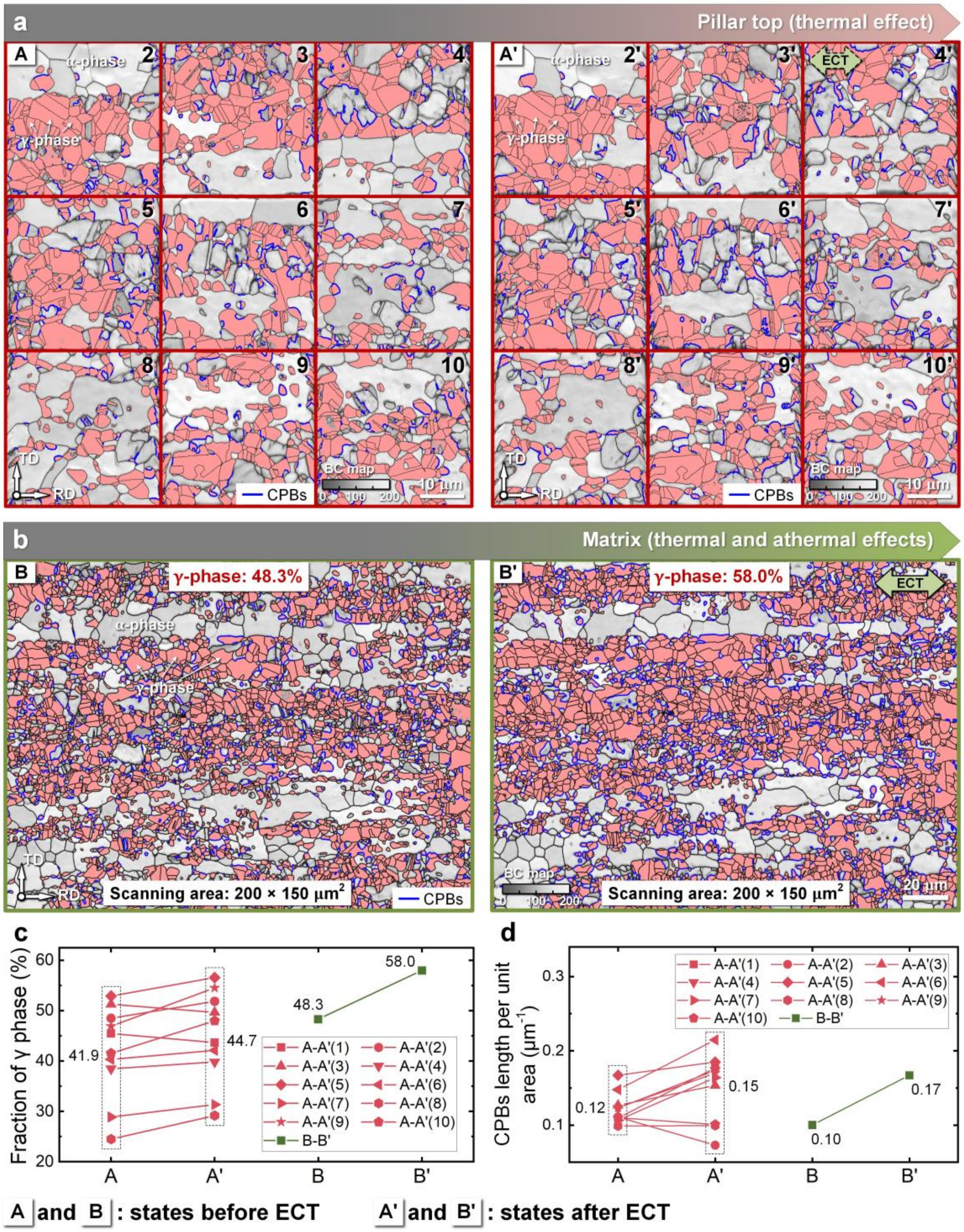

Supplementary Figure 3 | *In situ* EBSD observation on the additionally fabricated 9 pillars and the larger matrix region before and after pulsed ECT. (a–b) BC maps with the γ phase at the pillar top (No.2–No.10) and matrix before and after ECT, where the γ phase is shown in pink and the CPBs are shown in blue lines. **(c)** Changes in γ phase content. **(d)** Changes in CPBs length per unit area.

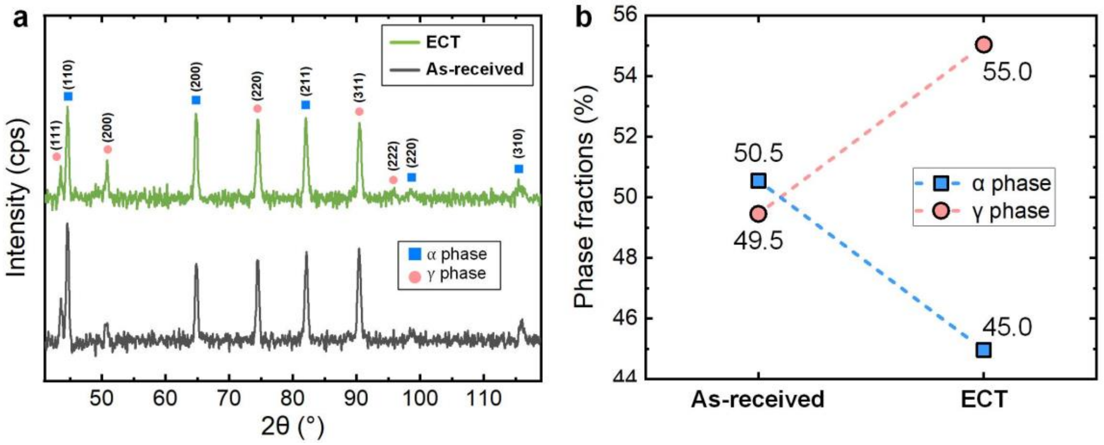

Supplementary Figure 4 | XRD analysis. (a) XRD profiles of the as-received and ECT-ed samples. **(b)** Phase fractions estimated based on the intensity of the diffraction peaks[1], I_{α} and I_{γ}.

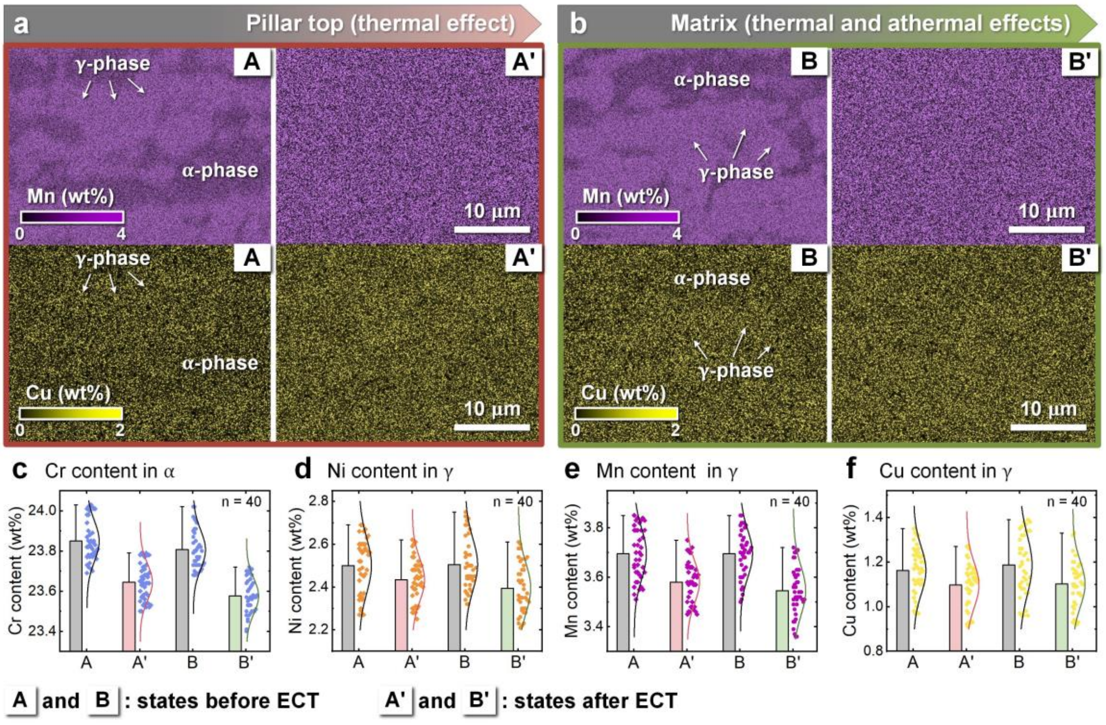

Supplementary Figure 5 | EDS analysis and statistical results. (a–b) Evolution of Mn and Cu distributions at the pillar top and matrix before and after ECT. **(c–f)** Statistical results of Cr, Ni, Mn, and Cu contents obtained from 40 sampling points, where the error bars are standard deviations.

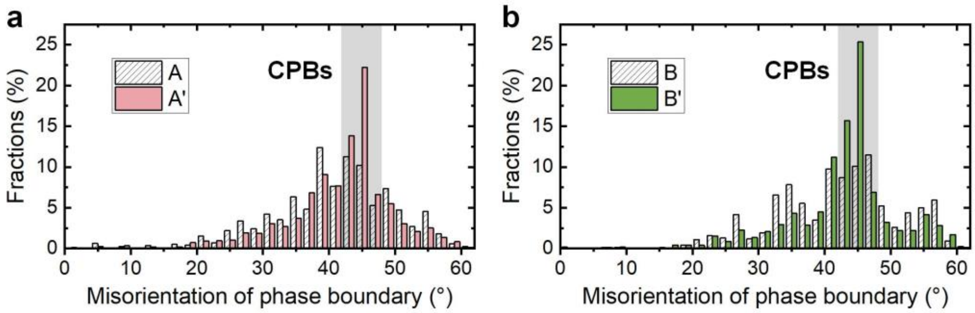

Supplementary Figure 6 | Misorientation distributions of the phase boundary (bcc-fcc) of the micromachined pillar and matrix before and after ECT. (a) Phase boundary distributions of the pillar top before (A) and after (A') ECT, where the CPBs are in the range of 42–47°. **(b)** Phase boundary distributions of the matrix before (B) and after (B') ECT.

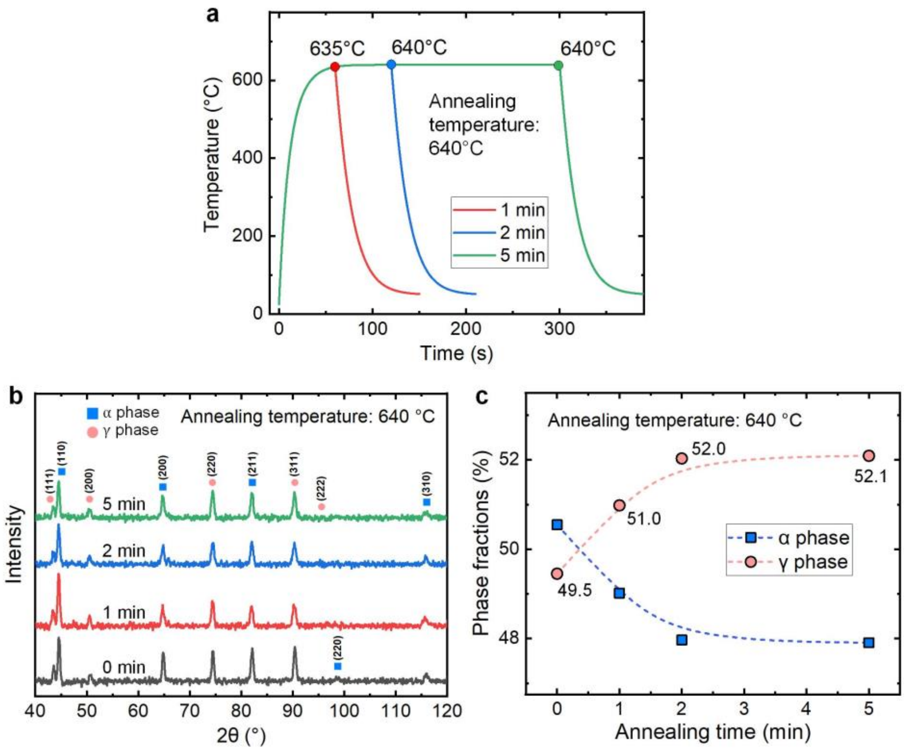

Supplementary Figure 7 | Analysis of rapidly annealed samples. (a) Temperature–time curves of the 1, 2, and 5 min annealed samples using a one-dimensional transient heat conduction model[2]. **(b)** XRD profiles of each sample. **(c)** Phase fractions estimated based on the intensity of the diffraction peaks[1], I_α and I_γ.

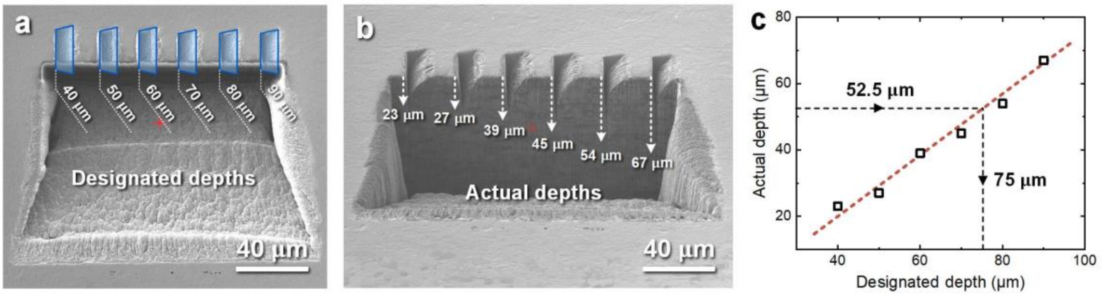

Supplementary Figure 8 | Determining the relationship between the designated and actual depths of the FIB process at an accelerating voltage of 30 kV and a current of 45 nA. (a–b) Top and side views of the FIB-processed trenches at different designated depths. **(c)** Relationship of the designated and actual depths. A 52.5-μm deep trench can be machined when the depth is set to approximately 75 μm.

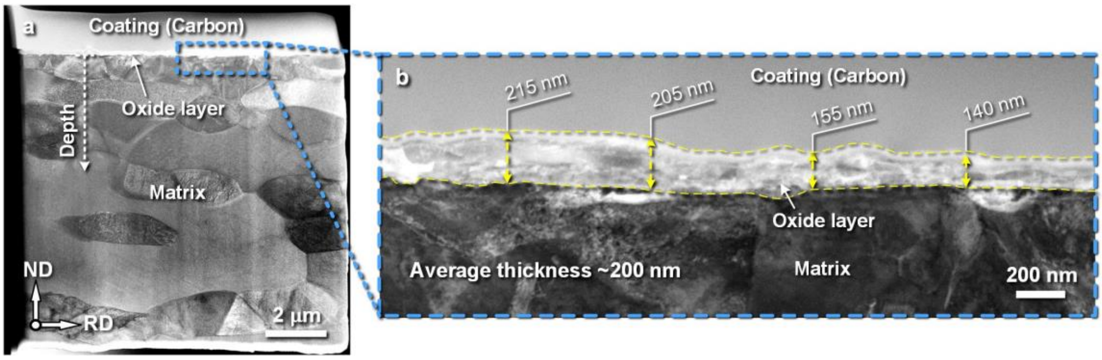

Supplementary Figure 9 | Thickness of the surface oxide layer after 20 pulses of ECT at 700 A mm^{-2} for 8 ms. (a) STEM image of the FIB processed TEM sample after ECT and **(b)** Local STEM image near the surface, demonstrating that the thickness of the oxide layer is ~200 nm.

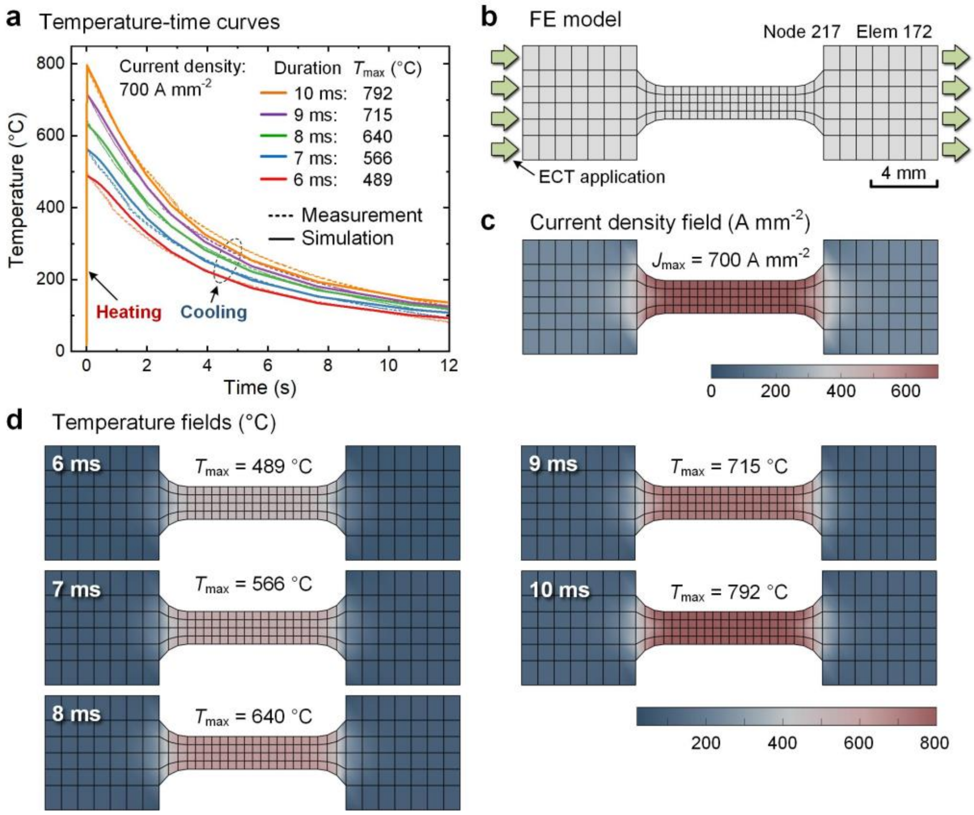

Supplementary Figure 10 | Temperature variations of the ECT-treated samples. (a) Temperature–time curves of the ECT-treated samples at 700 A mm^{-2} for 6–10 ms obtained from measurements and simulations and the maximum temperatures of each sample; **(b)** FE model of the sample; **(c)** Current density distribution, and **(d)** Temperature distributions of each sample with maximum temperatures marked near the images.

Supplementary Tables

Supplementary Table 1 Contents (wt%) of Cr, Ni, Mn, and Cu of the cantilever and matrix before and after ECT obtained through TEM-EDS.

	C/D (before ECT)	C' (after ECT)	D' (after ECT)
**Cr: α (γ)**	**24.17 ± 0.00**	**23.75 ± 0.00**	**23.96 ± 0.01**
	(20.42 ± 0.04)	(20.52 ± 0.01)	(20.69 ± 0.04)
**Ni: γ (α)**	**2.52 ± 0.01**	**2.44 ± 0.01**	**2.33 ± 0.00**
	(1.41 ± 0.01)	(1.51 ± 0.00)	(1.56 ± 0.00)
**Mn: γ (α)**	**4.18 ± 0.01**	**4.15 ± 0.01**	**4.11 ± 0.01**
	(3.44 ± 0.02)	(3.55 ± 0.01)	(3.58 ± 0.01)
**Cu: γ (α)**	**1.25 ± 0.00**	**1.14 ± 0.01**	**1.06 ± 0.00**
	(0.87 ± 0.01)	(0.90 ± 0.00)	(0.95 ± 0.01)

Supplementary Table 2 Relevant parameters for calculating Ni, Mn, and Cu diffusion from γ phase to α phase induced by ECT.

Parameters	Ni (γ→α)	Mn (γ→α)	Cu (γ→α)
Boltzmann constant (J K^{-1}): k	1.38×10^{-32}	1.38×10^{-32}	1.38×10^{-32}
Equivalent temperature (K): $T = T_{eff} = \int_0^{t_0} T(t)dt/t_0, (t_0 = 15s)$	595	595	595
Effective valence: Z^*	12.53 [3]	10.53 [3]	13.2 [3]
Elementary charge of a single electron (C): e	1.6×10^{-19}	1.6×10^{-19}	1.6×10^{-19}
Electrical resistivity (Ωm): ρ_r	8×10^{-7}	8×10^{-7}	8×10^{-7}
Current density (A m^{-2}): j_0	7×10^{8}	7×10^{8}	7×10^{8}
Mean grain size of α phase (μm): d_α	4.55	4.55	4.55
Mean grain size of γ phase (μm): d_γ	2.65	2.65	2.65
Diffusion distance (μm): $x = (d_\alpha + d_\gamma)/2$	3.6	3.6	3.6
Pulse number: n_p	20	20	20

Supplementary Notes

1. FE simulation

The FE simulation performed in this study involved electrostatic field and thermal analyses. Additionally, the models of the pillar and cantilever were simplified into a two-dimensional strain problem, as illustrated in Supplementary Figure 11a and 11b. The models were partitioned using quadrilateral elements, and the simulation program was developed and executed using MATLAB. A brief description of the governing equations and boundary conditions at Γ_1, Γ_2, and Γ_3 is presented below.

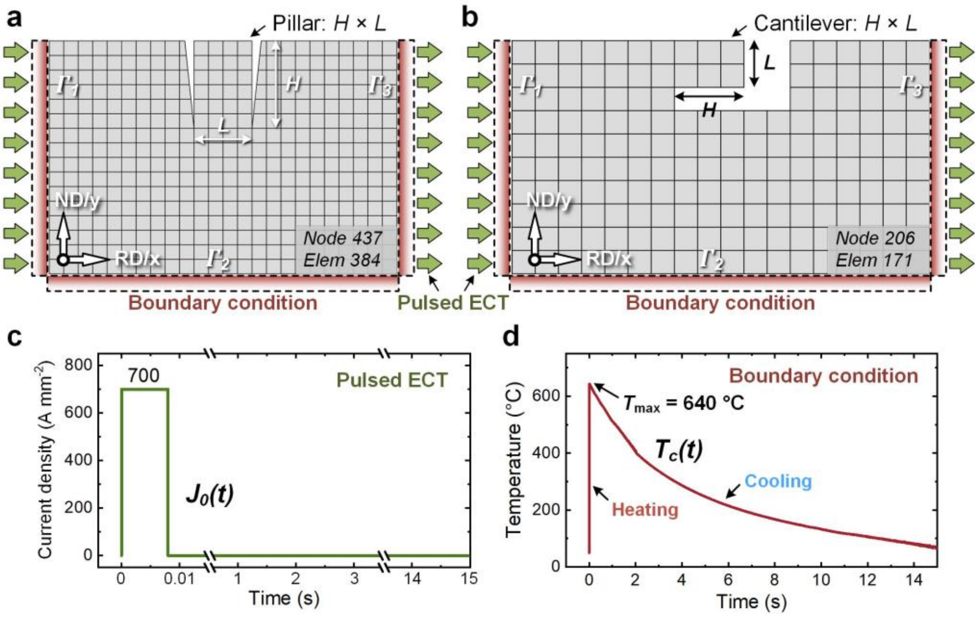

Supplementary Figure 11 | Two-dimensional FE models of the micromachined pillar and cantilever. (a–b) FE models of the pillar and cantilever with a size of $H \times L$ and ECT applied along the horizontal with thermal boundary conditions. **(c)** Pulsed ECT with a constant current density of 700 A mm^{-2} for 8 ms. **(d)** Boundary condition (thermal history of the ECT-treated sample) obtained from thermal sensors.

1. Electrostatic field analysis

The governing equation of the static electric problem is based on the conservation equation of current density, Ohm's law, and the relationship between electric field and electric potential[4,5], as shown below:

$$\nabla \cdot \mathbf{J} = 0 \quad (1)$$

$$\mathbf{J} = \sigma_{EC}\mathbf{E} \quad (2)$$

$$\mathbf{E} = -\nabla \Phi \quad (3)$$

where ∇ represents the Nabla operator ($\nabla = \partial/\partial x\mathbf{i} + \partial/\partial y\mathbf{j}$); $\mathbf{J}$, σ_{EC}, $\mathbf{E}$, and Φ denote the current density, electric conductivity, electric field, and electric potential, respectively.

The boundary conditions can be mathematically expressed as:

$$\begin{cases} \nabla\phi|_{\Gamma_1,x} = -J_0(t)/\sigma_{EC} \\ \nabla\phi|_{\Gamma_3,x} = 0 \end{cases} \quad (4)$$

where $J_0(t)$ represents the current density applied along the rolling direction (or x-axis), as shown in Supplementary Figure 11c.

2. Thermal analysis

The governing equation of the transient heat transfer problem includes the energy stored in the control volume, thermal conduction, thermal convection (energy dissipation), and generation of thermal by Joule heating[5,6]:

$$c\rho_D t_n \frac{\partial T}{\partial t} = -\nabla \cdot \mathbf{q}t_n + q_{conv} + q_{gene}t_n \quad (5)$$

$$\mathbf{q} = -k\nabla T \quad (6)$$

$$q_{conv} = -2h(T - T_0) \quad (7)$$

$$q_{gene} = \frac{|\mathbf{J}|^2}{\sigma_{EC}} \quad (8)$$

where c, ρ_D, t_n, T, t, $\mathbf{q}$, q_{conv}, q_{gene}, k, h, and T_0 are the specific heat coefficient, density, thickness of the sample, temperature, time, flux of thermal conduction, flux of thermal convection, thermal flux generated by the electric current, thermal conductivity coefficient, thermal convection coefficient, and initial temperature (25 °C), respectively.

For micromachined structures, the thermal boundary condition is thermal variations ($T_c(t)$) in the matrix material under ECT (measured using thermal sensors), as shown in Supplementary Figure 11d, and the corresponding equation is:

$$T|_{\Gamma_1\,\&\,\Gamma_2\,\&\,\Gamma_3} = T_c(t) \quad (9)$$

2. Simple heat conduction model of the micromachined structures

In this study, we simplified the micromachined structures under the influence of electric current into a one-dimensional heat transfer problem, as depicted in Supplementary Figure 12. For the micromachined pillar, the TCP extends from the base to the top of the pillar, whereas for the micromachined cantilever, the path includes a bending section. Supplementary Figure 12c presents the simple heat conduction model of the micromachined structure; the left side is the matrix (labeled as 1), while the right is the interconnected micromachined structure (labeled as 2) with a cross-sectional area A and length h.

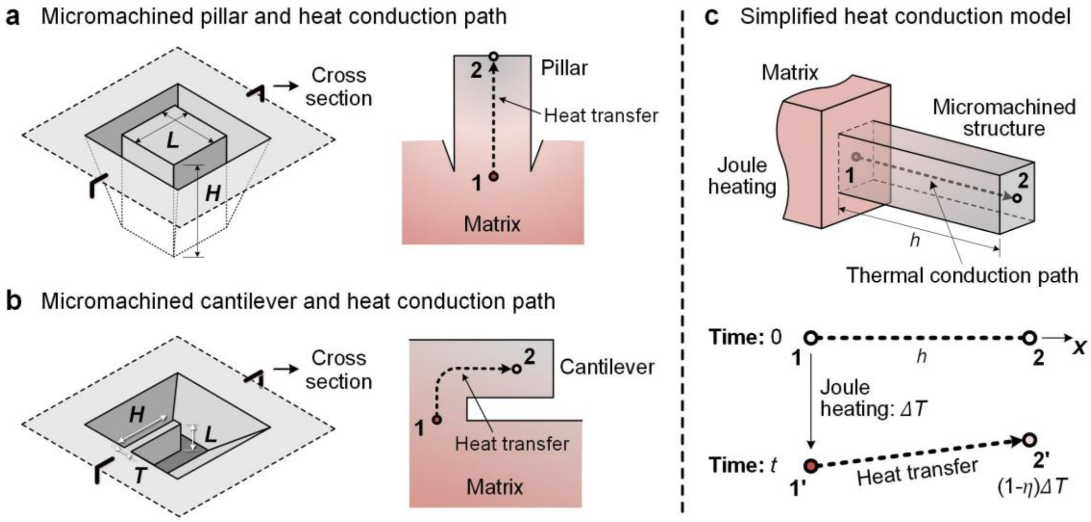

Supplementary Figure 12 | Simple heat conduction model of the micromachined structure. (a–b) Schematic of the micromachined pillar and cantilever with heat transfer from the matrix to micromachined structures and **(c)** simple heat conduction model.

The matrix rapidly heats up due to Joule heating with an increase of $\Delta T = j_0^2 t / c\rho\sigma$[7], where j_0 and t are the current density and duration of the pulsed ECT, respectively; and c, ρ, and σ are the specific heat capacity, density, and electrical conductivity of the material, respectively. The micromachined structure experiences forced heating due to heat conduction from the matrix. Hence, the temperature of the micromachined structure remains lower than that of the matrix. To ensure that the temperature at the end of the micromachined structure does not decrease significantly, the length of the TCP must be limited. Assuming a temperature decrease

rate of η, the temperature increase at the end of the micromachined structure is $(1-\eta)\Delta T$. For the one-dimensional heat conduction problem, assuming the thermal conductivity of the material is isotropic and uniform, the temperature distribution along the micromachined structure is linear[6]. Consequently, the average temperature rise across the micromachined structure is $(1-\eta/2)\Delta T$. Therefore, the stored energy is,

$$Q_s = cm\left(1-\frac{\eta}{2}\right)\Delta T = c\cdot\rho Ah\cdot\left(1-\frac{\eta}{2}\right)\frac{j_0^2 t}{c\rho\sigma}\ , \qquad (10)$$

where m is the mass of the micromachined structure, which can be expressed as $m=\rho Ah$.

Therefore, the energy stored in the micromachined structure is conducted from the matrix through the cross section at position 1, which is expressed as $Q_t = q_x At$. q_x represents the heat flux along the length direction of the micromachined structure[6], which can be expressed as $q_x = -k\cdot dT/dx$, where k is the thermal conductivity. Therefore, during the application of ECT for a duration of t, the heat transferred to the micromachined structure is:

$$Q_t = \int_0^t kAt\frac{j_0^2}{c\rho\sigma h}dt = \frac{kAj_0^2t^2}{2c\rho\sigma h}\ . \qquad (11)$$

Assuming $Q_t = Q_s$, we get,

$$h = \sqrt{\alpha t/(2-\eta)}\ , \qquad (12)$$

where α represents the thermal diffusivity, which can be expressed as $\alpha = k/c\rho$.

Supplementary References

1. Gu, S. *et al.* Realizing strength–ductility synergy in a lean duplex stainless steel through enhanced TRIP effect via pulsed electric current treatment. *Mater. Sci. Eng. A* **883**, 145534 (2023).
2. Gu, S. *et al.* Ultrahigh deformability of Ti-6Al-4V assisted by high-density pulsed electric current treatment. *J. Alloy Compd.* **973**, 172892 (2024).
3. 1.1.2: Effective Nuclear Charge. *Chemistry LibreTexts* https://chem.libretexts.org/Courses/Saint_Marys_College_Notre_Dame_IN/CHEM_342%3A_Bio-inorganic_Chemistry/Readings/Week_1%3A_Analysis_of_Periodic_Trends/1.1%3A_Concepts_and_principles_that_explain_periodic_trends/1.1.2%3A_Effective_Nuclear_Charge (2019).
4. Cheng, D. K. *Field and Wave Electromagnetics*. (Addison-Wesley, Boston, 1989).
5. Zienkiewicz, O. C. & Taylor, R. L. *The Finite Element Method*. (Butterworth-Heinemann, Boston, 2000).
6. Bergman, T. L., Lavine, A. S. & Incropera, F. P. *Fundamentals of Heat and Mass Transfer, 7th Edition*. (John Wiley & Sons, Hoboken, 2011).
7. Zhou, Y., Xiao, S. & Guo, J. Recrystallized microstructure in cold worked brass produced by electropulsing treatment. *Mater. Lett.* **58**, 1948–1951 (2004).
